# Factors influencing the intention to purchase health insurance: a study of Indian tobacco and alcohol consumers

**DOI:** 10.3389/fpubh.2024.1332511

**Published:** 2024-03-15

**Authors:** Ashok Mishra, Mohammed Jamshed, Asad Ahmad, Swati Garg, Dag Øivind Madsen

**Affiliations:** ^1^Department of Management, Jamia Hamdard, New Delhi, India; ^2^USN School of Business, University of South-Eastern Norway, Kongsberg, Norway

**Keywords:** consumer behavior, factor analysis, health insurance, theory of planned behavior, intention to purchase, India

## Abstract

**Introduction:**

This study empirically investigates the attitude of tobacco and alcohol consumers towards health insurance purchase in India. The study aims to determine the factors which plays a significant role in determining the purchase intention of health insurance among tobacco and alcohol consumers.

**Methods:**

We propose an extended theory of planned behavior (TPB) model comprising factors like attitude, subjective norms, perceived behavior control, perceived usefulness, perceived product risk, and intention to purchase. We collected responses from 420 tobacco and alcohol consumers through a Google Form link shared via different social media platforms. SPSS has been used to perform exploratory factor analysis, whereas AMOS has been used to validate the constructs, confirm the relationships among the variables, and analyze the data.

**Results:**

The analysis outcomes demonstrate that subjective norms, perceived product risk, and perceived behavioral control are the factors that have a positive and significant effect on health insurance purchase intention among consumers.

**Discussion:**

This research offers valuable insights to the insurance sector, government officials, policymakers, and academicians. Insurance companies may consider the criteria analysed when creating policies to promote the expansion of the health insurance sector.

## 1 Introduction

There are around 1.3 Billion tobacco users globally; out of them, an estimated 80% of tobacco users reside in low-and middle-income nations (1). By spendthrift of income from essentials like food and shelter, tobacco and alcohol consumers are found to push families into greater debt. Additionally, it results in the early demise and disability of working-age individuals within homes, which lowers household income and raises healthcare expenses. In India, health insurance is rapidly rising as a prominent sector, following life and automobile insurance. The health insurance industry is a primary driver of the expansion of the general insurance sector. It contributes around 29% of India’s total general insurance premium income (2). India spends 3.01% of its gross domestic product (GDP) on health, but only a third of it comes from public funds, a proportion lower than that of other developing countries (3). In the advent of decreasing saving rates from 29.9% (2020) to 28.2% (2021) in India (4), people generally take loans from organized and unorganized resources when they get ill and repay the same when they get healthy again. Health insurance came into existence to support consumers and prevent such situations. The primary goal of health insurance policy is to reduce the risk people face in the event of unexpected healthcare costs, preventing them from being negatively impacted by financial burdens.

Health insurance denotes the source of funds for medical care financing, in which one decides to purchase healthcare insurance on a specified contract (5). Health insurance is cited as a crucial intervention in a recent government of India policy statement to give financial security and lower out-of-pocket (OOP) expenses. According to reports, 70% of the population might be covered by existing health insurance programs, including government-subsidized plans, social health insurance programs, and private voluntary health insurance (6). In terms of both employment and revenue, the healthcare industry has grown to be one of India’s biggest industries, and it is in the top three in terms of incremental growth. Rising income levels, increased health awareness, the prevalence of lifestyle diseases, and government initiatives like the Pradhan Mantri Jan Arogya Yojana (PM-JAY), National Digital Health Mission (NDHM), etc. are all expected to contribute to the healthcare market in India reaching an estimated $8.6 trillion in value by 2022 (7).

Health insurance is increasingly vital in health care services (8). In 2018, India launched one of the biggest government-funded health insurance schemes to protect the vulnerable population from financial hardship and encourage them to seek hassle-free health care (9). Health insurance programs play a significant role in fighting poverty as they also connect to other social supports such as food assistance. Children’s health insurance programs are linked to decreasing mortality, fewer chronic health conditions, better educational attainment, and less reliance on government support (10). A related study has shown that a mother’s health insurance subscription influences child stunting and underweight through maternal healthcare utilization and providing diversified diets to the children (11).

With 1.35 billion beneficiaries, India’s healthcare system is one of the world’s largest, but most citizens lack access to health coverage or quality care (12). In India, there are 267 million tobacco users, with one of the world’s highest rates of tobacco use. It has been reported that tobacco causes a substantial economic burden, totaling an estimated USD 22.4 billion annually (13). In 2018, the World Health Organization (WHO) estimated that tobacco use accounted for one million deaths, or 9.5% of all deaths in the country. Notably, more than 3,500,000 deaths annually are associated with smokeless tobacco use. Furthermore, alcohol consumption in India amounted to about five billion liters in 2020 and is estimated to reach about 6.21 billion liters by 2024. According to a study, around 88% of Indians aged under 25 purchase or consume alcohol (14). According to the National Crime Records Bureau (NCRB), 6,172 people died between 2016 and 2020 due to the consumption of illicit liquor in India (15). Such consumers cover a large portion of the young population and the nation’s future, making it necessary to cover them under health insurance study. The country’s healthcare system has not been able to respond enough to the challenge. In 2018, India spent roughly 1.3% of its GDP on health care, one of the lowest health expenditures globally (3).

Few studies on the Theory of Planned Behavior (TPB) have been undertaken in India, focusing on insurance sectors. Still, we could not find studies focusing on the tobacco and alcohol consumers of India. However, the literature addressing the purchase intention of health insurance is also limited. The present study aims to examine the factors affecting the purchase of health insurance among tobacco and alcohol consumers. The study empirically investigated how attitude toward behavior, subjective norms, perceived behavior control, perceived usefulness, perceived product risk, intention to purchase, and behavioral control may play a significant role in determining the purchase of health insurance among tobacco and alcohol consumers in the context of emerging countries like India.

The primary purpose of this research is to explore and determine the behavioral intentions of alcohol and tobacco consumers toward purchasing health insurance in India. Understanding the behavioral intention of Indians may provide a useful framework for future marketing communications to reach the target population and determine ways to encourage more Indians to consider buying health insurance. The findings will likely help health insurance marketers form their strategic focus in developing the health insurance market in India. Applying planned behavior to health insurance services in India will contribute to the current literature by analyzing the underlying constructs of the behavioral intention toward purchasing health insurance in a developing country.

## 2 Theoretical background and hypotheses development

The effectiveness of the TPB in predicting the intention to purchase various products and services has been well-established by several researchers worldwide. The theory connects beliefs and behavior. This model was postulated by Ajzen (16), which states that an individual’s behavior in purchasing a product or service is determined by their intention. The intention to purchase a product/service depends upon the readiness of a consumer and how well they perceive the product. According to (17), purchase intention can be described as the consumer disposition to buy a product. Previous studies have stated that purchase intention is a base for actual purchase behavior (17–19). Fishbein and Ajzen (20) state that actual purchase behavior is influenced by the desire to purchase that product.

**Figure 1:**
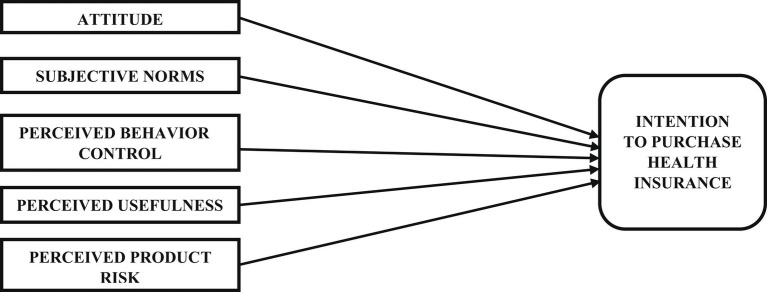
Proposed model.

Considering the TPB’s robustness, several researchers have used the model in different contexts (18, 21, 22). In the present study, we have used an extension of the TPB model (Figure 1) to predict alcohol and tobacco consumers’ behavior toward buying health insurance. In this paper, the relationship among Attitude toward behavior (ATB), Subjective norms (SN), Perceived behavior control (PBC), Perceived usefulness (PU), Perceived product risk (PPR), with Intention to purchase has been explored.

### 2.1 Attitude toward behavior and intention to purchase

The term “attitude” refers to a psychological inclination expressed by evaluating something with either some degree of favor or disapproval (23). Attitude is how an individual experiences things and makes conceptions that influence their behavior (24). According to Fishbein and Ajzen (20), an attitude toward behavior indicates whether an individual likes or dislikes a particular thing. The intention to participate in an activity is stronger for individuals with a positive attitude toward it (25). A consumer is far more likely to purchase an insurance product to satisfy their consumption demands if their attitude is positive. Guan et al. (26) reported that attitude is an important factor influencing consumer buying decisions toward insurance products. The TPB has been used in several types of research to evaluate the relationship between consumers’ attitudes and intentions to buy goods and services (23, 27–29). Based on the above literature, the following hypothesis is framed:

*H1*: Attitude has a positive effect on the purchase intention of health insurance among tobacco and alcohol consumers.

### 2.2 Subjective norms and intention to purchase

Ajzen (16) describes subjective norms as being related to a person’s perception of social pressure to engage in a particular action. According to Lujja et al. (30), a person’s perception of subjective norms depends on whether they feel that others who are significant to them should engage in a particular conduct. The opinion of one’s friends, family, and peers might impact people’s purchase intention. Subjective norms play an important role and positively influence consumption patterns (31). In social science research, it has been widely accepted that subjective norms are a predictor of intention (29, 32). Researchers like Jain (22) have suggested that subjective norms are important in determining consumers’ purchase intention. Several researchers have suggested a positive and significant relationship between subjective norms and intent to purchase (19, 33, 34). Based on the above literature, the following hypothesis is framed:

*H2*: Subjective norms positively impact the purchase intention of health insurance among tobacco and alcohol consumers.

### 2.3 Perceived behavior control and intention to purchase

The perspective of an individual toward the ease or difficulty of carrying out any behavior is known as perceived behavioral control (34, 35). Perceived behavior control, directly and indirectly, impacts consumer behavior through intention, assuming that this component has motivational consequences for behavioral intention (32). Moreover, the consumer perception and their ability to engage in a specific activity directly influence their purchase intention. If consumers feel they have the necessary tools and the confidence to carry out a behavior, they are more inclined to engage in that behavior. Husin and Ab Rahman (35) stated that perceived behavioral control plays a significant role in the consumer buying decision and positively relates to their intention to purchase. Furthermore, several researchers have shown the significant effect of perceived behavior control on purchase intention in their studies (19, 36). Based on the above literature following hypothesis is framed:

*H3*: Perceived behavior control has a positive effect on the purchase intention of health insurance among tobacco and alcohol consumers.

### 2.4 Perceived usefulness and intention to purchase

It is stated that increasing a specific utility in people’s perceptions will make them feel more favorable about buying a particular product or service (37). The perceived usefulness of any insurance impacts a person’s decision to buy that particular insurance (23). Perceived usefulness significantly impacts how people feel about buying health insurance (38). In their study, Aziz et al. (17) highlighted a positive correlation between the purchase of health insurance and consumer intention. Moreover, they have suggested that insurance firms should consider and communicate its usefulness to motivate consumers to get health insurance. Several studies have advocated the significant impact of perceived usefulness on purchase intention (21, 39). Thus, the following hypothesis is framed:

*H4*: Perceived usefulness has a positive effect on the purchase intention of health insurance among tobacco and alcohol consumers.

### 2.5 Perceived product risk and intention to purchase

Perceived risk is defined as the expectation of unfavorable consequences made by the customer (40, 41). Product risk refers to the probability that a purchased product will not offer the intended advantages or function properly (42, 43). Product risk involves a potential loss if the product does not meet consumer expectations regarding product standards and quality (44, 45). Perceived risk plays a significant role in identifying consumer perception and intention to purchase (44). Consumer intention toward purchasing health insurance depends greatly on the perceived risk (Liebenberg et al., 2012). Perceived risk has been suggested to be an important factor in determining the consumers’ behavioral intention. Several researchers have found a significant impact of perceived risk on the behavioral patterns of consumers (44–46). Based on the above literature, the following hypothesis is framed:

*H5*: Perceived product risk has a positive effect on the purchase intention of health insurance among tobacco and alcohol consumers.

## 3 Research methodology

### 3.1 Stage 1: scale development

This study is an extension of the TPB model to investigate the intention to purchase health insurance among tobacco and alcohol consumers in India. Taking a cue from the extant literature, a model comprising of factors like attitude toward behavior (ATB), subjective norms (SNorm), perceived behavior control (PERBC), perceived usefulness (PU), perceived product risk (PPRsk), and intention to purchase health insurance (INHI) was proposed. The study scale comprised 30 items, a six-factor scale (Table 1). The items used in the study were adapted from the work of Mamun et al. (23) and Brahmana et al. (21). The questionnaire items measuring all the constructs were based on a *5-point* Likert scale (1 = *strongly disagree;* 2 = *disagree;* 3 = *neither agree nor disagree; 4 = agree;* 5 = *strongly agree*).

**Table 1:** Exploratory factor analysis.

Code	Items	Loadings	Decision	Reliability
	Attitude			
ATT1	I think that buying health insurance is a good choice	<4.0	Not Retained	
ATT2	I think buying health insurance is essential for every one	Cross	Not Retained	
ATT3	I think buying health insurance should be compulsory	<4.0	Not Retained	
ATT4	I will recommend health insurance to other	Cross	Not Retained	
ATT5	I think buying health insurance is valuable	Cross	Not Retained	
	Subjective Norms			
SN1	People will like it if I purchase health insurance	<4.0	Not Retained	
SN2	My social groups think I should purchase health insurance	0.621	Retained	0.936
SN3	My friends/external parties share important ideas about purchasing health insurance	0.824	Retained	
SN4	People who influence my decision think that I should purchase health insurance	0.797	Retained	
SN5	People whose opinion I value think that I should purchase health insurance	0.723	Retained	
	Perceived Behavioral Control			
PBC1	I can handle any (money, time, information related) difficulties associated with my buying decision	0.657	Retained	0.892
PBC2	I can buy health insurance comfortably well on my own	0.808	Retained	
PBC3	I have sufficient knowledge to purchase health insurance	0.817	Retained	
PBC4	I can purchase health insurance without any help from other	0.862	Retained	
PBC5	I know different sources from where to buy health insurance	0.747	Retained	
	Perceived Usefulness			
PU1	Purchasing health insurance enables me to ease my future expenses	Cross	Not Retained	
PU2	Purchasing health insurance improves my health benefits	Cross	Not Retained	
PU3	Using health insurance policy will improve my performance in handling my financial needs	Cross	Not Retained	
PU4	Purchasing health insurance policy will enhance my Financial dependency	<4.0	Not Retained	
PU5	Purchasing health insurance will be useful to cater critical illness case	Cross	Not Retained	
	Perceived Product Risk			
PPR1	I am unsure whether I can get desired protection from the insurance policy	0.749	Retained	0.935
PPR2	I am afraid that insurance will create unnecessary problems at the time of claim	0.851	Retained	
PPR3	Failure to pay the premium on time, I am afraid to face (Penalty/Scheme Collapse)	0.780	Retained	
PPR4	I am unsure that insurance company will settle my claim on time	0.847	Retained	
PPR5	I am unsure about the help the insurance company will offer to my family in my absence	0.808	Retained	
	Intention to Purchase Health Insurance			
IHI1	I will make effort to purchase health insurance in the future	0.660	Retained	0.899
IHI2	I will plan for health insurance in the future	0.763	Retained	
IHI3	I would only buy health insurance when I am convinced about its benefits	0.738	Retained	
IHI4	The moment I know the value of Health insurance I will purchase it as soon as possible	0.772	Retained	
IHI5	I am expected to purchase health insurance in the future	0.831	Retained	
*KMO: 0.939*				

### 3.2 Stage 2: pilot study

To check the one-dimensionality of the scale, researchers have suggested going for a pilot survey before the actual data collection (47). In the present study, we carried out a pilot survey on the proposed model to check the one-dimensionality of the used scale. The initial data was collected from 85 respondents and further analyzed using exploratory factor analysis (EFA) on SPSS 22. The Bartlett’s Test of Sphericity (BTS) was found to be significant. The Kaiser-Meyer-Olkin (KMO) value was also found to be satisfactory (0.939; >0.6) (48). The total variance was found to be 77.46.

Considering the previous research suggestions, the current study also looked at factor loadings and communalities to decide which items should be kept and which should be deleted. Some items were removed due to cross-loading or low factor loadings of less than 0.5. Because of low loadings (<0.5) and cross-loadings, certain items of the proposed scale had to be dropped. The iterative process of scale refinement resulted in a shorter four-factor (19-item) scale comprising subjective norms, perceived behavior control, perceived product risk, and intention to purchase health insurance (Table 1). It can be surmised from the analysis that the process resulted in the dropping of all the items of Attitude and Perceived Usefulness because of low as well as cross-loadings. Further, the hypotheses H1 and H4 had to be dropped. Also, one item of subjective norm had to be dropped because of low loading. The factors of the scale were found to reliable, with the alpha value was observed to be in the acceptable range, i.e., SN- 0.936, PBC- 0.892, PPR- 0.935, and IHI- 0.899 (48).

### 3.3 Data collection and ethical considerations

It has been recommended that the sample size of a study must be 10 times the number of items used in a scale (49). This means that 19 * 10 = 190 was the appropriate sample size for the present work. We gathered participant feedback using digital (via a link to a Google Form) and traditional methods (using paper and pencil). As per the suggestion of Bertilsson (50), if the respondents are offered anonymity in a study, no informed consent is required from them. In the present study, we have stood up to the ethical guidelines by obtaining prior consent from the respondents and maintaining the respondents’ anonymity in the research.

With the constraints of having no exact data on the tobacco and alcohol consumers, convenience sampling has been used for the study. The convenience sampling method is adopted because it is a type of nonprobability or non-random sampling in which study participants are selected based on practical considerations such as proximity, ease of accessibility, availability at a particular time, or willingness to participate. This sampling method is affordable and accessible, and the respondents are readily available (51). The study survey has been conducted in the Delhi NCR region. Delhi is also teeming with diverse socioeconomic classes (52). The largest metropolitan region in the nation is also located in the study area. It draws a sizable number of migrants from different states (53, 54). Over a three-month period, 420 replies were received in total, which was more than the minimal number of suggested responses needed. Out of the 420 responses, 389 were deemed suitable for subsequent analysis. The researchers excluded responses with irregular answering patterns or incomplete data on the variables in question. The sample individuals were between the age group of 18–50 who, according to researchers, have been found to consume more tobacco and alcohol compared to others (55, 56). Over 85% of the responses came from a younger demographic, specifically individuals aged between 18 and 30 years. The questionnaires focused on tobacco and alcohol use; therefore, respondents under the age of 18 were excluded from the study in compliance with Indian government regulations that categorize them as minors COPTA (57). The demographic profile of the respondents is presented below in Table 2.

**Table 2:** Demographic table.

Items	Classification	Frequency	Percentage
Gender	Male Female	317 72	81.49% 18.51%
Age	18–30 31–40 41–50 51 above	332 44 8 5	85.34% 11.31% 2.06% 1.28%
Marital status	Married Unmarried	77 312	19.79% 80.21%
Education	Below Graduation Above Graduation	56 333	14.40% 85.60%
Occupation	Salaried Business Unemployed	232 40 117	59.64% 10.28% 30.08%
Annual Income	No income Below 3 lakhs 3–5 lakh 5–10 lakh Above10 lakhs	99 110 89 57 34	25.45% 28.28% 22.89% 14.66% 8.73%
Type of family	Nuclear Joint	274 115	70.43% 29.57%
Residence area	Urban Rural	300 89	77.10% 22.90%

## 4 Data analysis

**Figure 2:**
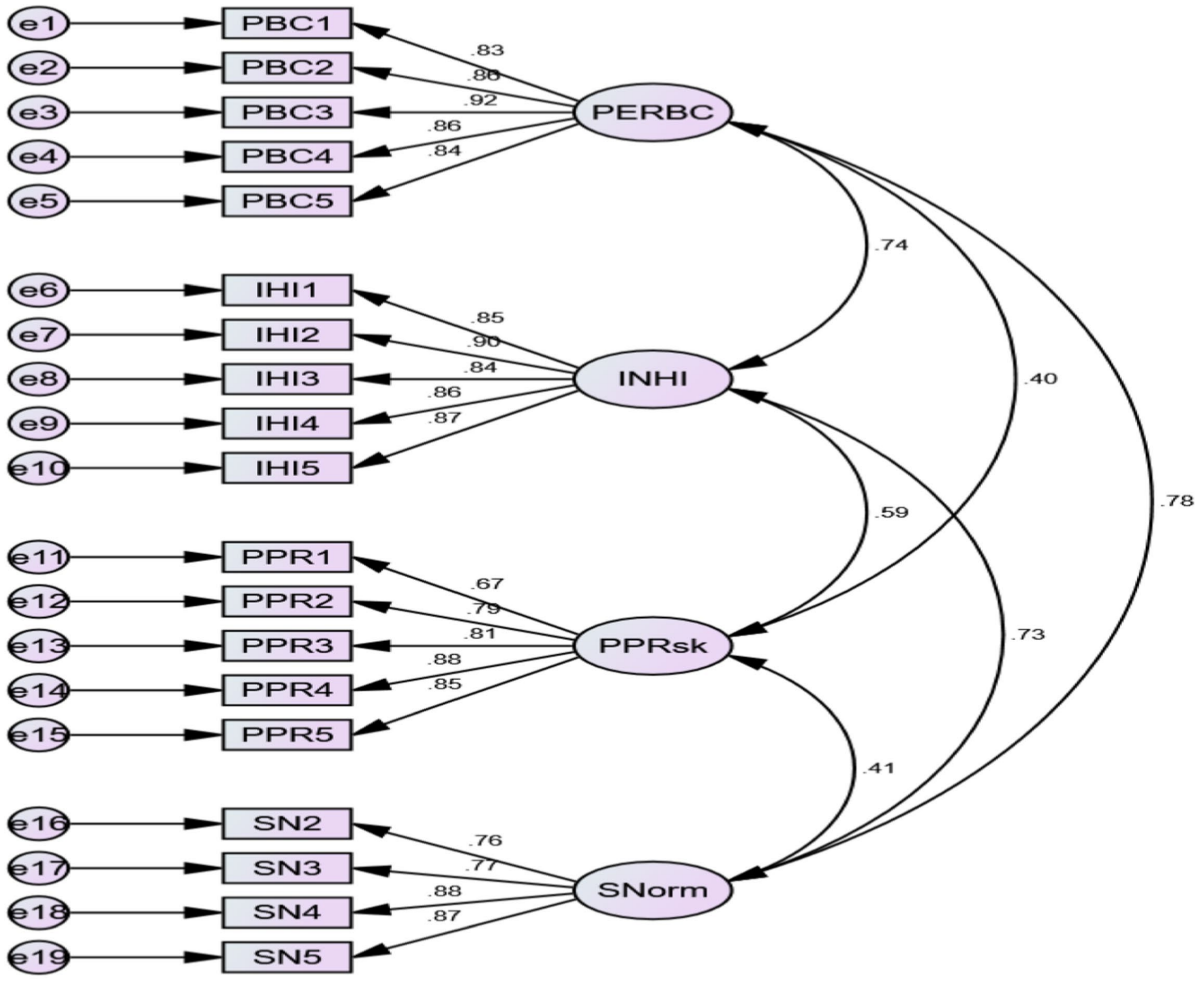
Measurement model. Perceived Behavior Control (PERBC), Intention to Purchase Health Insurance (INHI), Perceived Product Risk (PPRsk), Subjective Norms (SNorm).

Confirmatory factor analysis has been performed using AMOS on the four factors, i.e., perceived behavior control (PERBC), intention to purchase health insurance (INHI), perceived product risk (PPR), and subjective norms (SN). To consider the specification of the items loading on their respective variables, the measurement model (Figure 2) was evaluated for model fit. The loadings of the items with their corresponding variables were found to be within the acceptable range. The measurement model’s fit indices indicated a good model fit with comparative fit index (CFI) = 0.942, the goodness of fit index (GFI) = 0.886, adjusted goodness of fit index (AGFI) = 0.852 and root mean square error of approximation (RMSEA) = 0.81. Although the value of GFI and AGFI is less than 0.90 researchers have stated that these standards can be regarded as acceptable (58, 59). Thus, the overall model was found to be satisfactory.

Convergent and discriminant validity were examined using the average variance extracted (AVE) (60). The AVE for the current measurement model was determined to be above the benchmark value of 0.5 (Table 3), indicating good convergent validity (60, 61). Each construct’s square root of AVE (bold diagonal values in Table 3) was greater than the inter-construct correlations, showing that the scale’s discriminant validity was satisfactory (60, 61). Researchers like Hair et al. (60) and Malhotra and Dash (48), have suggested the reliability of the factors to be demonstrated by the composite reliability, which was significantly higher than the threshold value of 0.7 (Table 3). Thus, the values suggest the scale to be reliable and valid.

**Table 3:** Validity and reliability.

	CR	AVE	MSV	MaxR(H)	PERBC	INHI	PPR	SN
PERBC	0.936	0.744	0.605	0.940	0.863			
INHI	0.936	0.745	0.552	0.938	0.743***	0.863		
PPR	0.900	0.645	0.344	0.912	0.402***	0.586***	0.803	
SN	0.894	0.680	0.605	0.905	0.778***	0.734***	0.411***	0.825

PERBC (perceived Behavior Control), INHI (Intention to Purchase Health Insurance), PPR (Perceived Product Risk), SN (Subjective Norms). *** Inter-construct correlations.

### 4.1 Structural model

**Figure 3:**
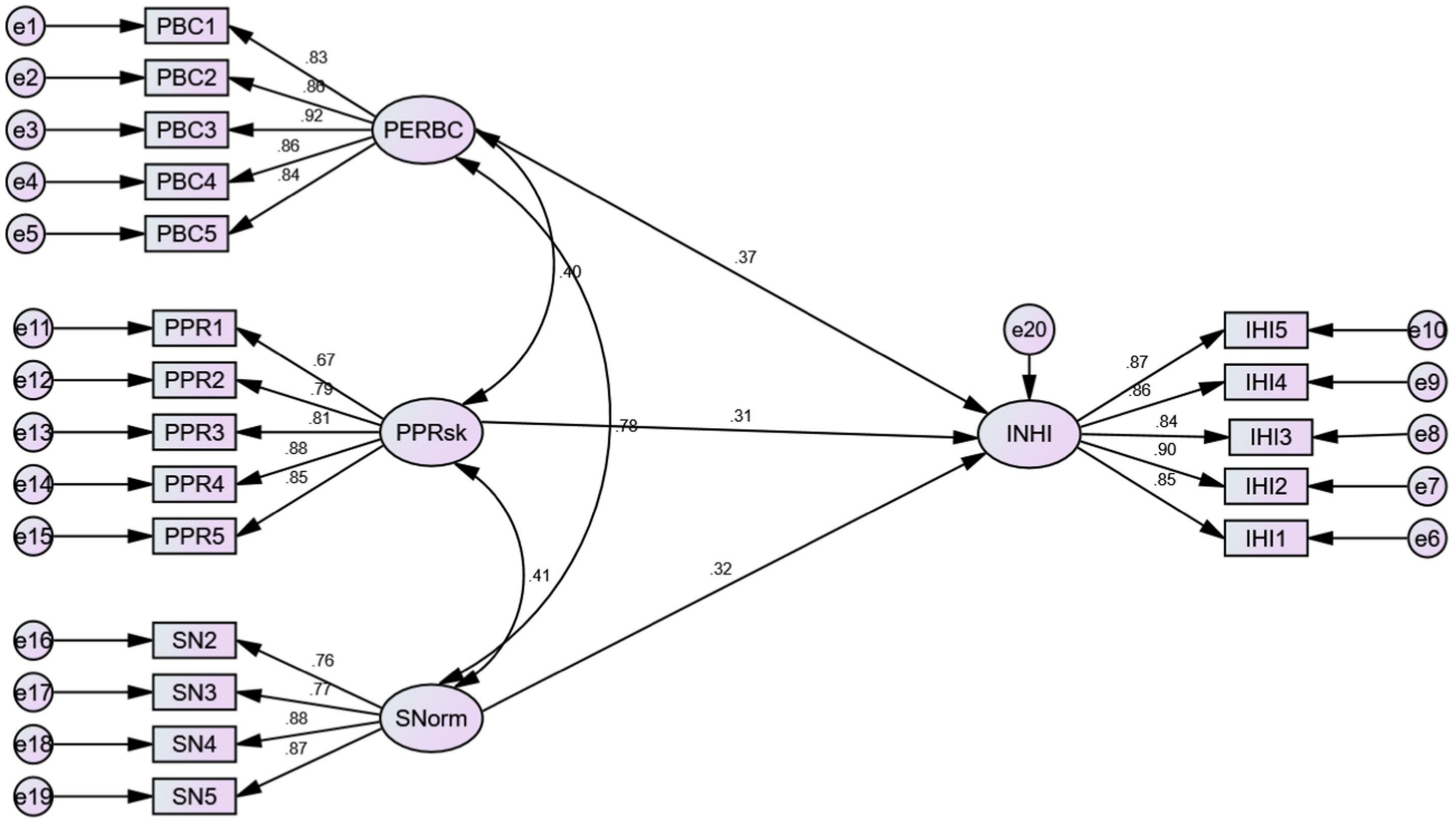
Structural model.

Table 4 presents model fit indicators of the structural model (Figure 3). With CMIN/df = 4.194 and RMSEA = 0.81, the CFI value was found to be greater than that the recommended value of 0.90 The GFI and AGFI values were less than 0.90, but researchers have suggested values below that too can be regarded fine (64). Thus, the goodness-of-fit measures and overall model fit indices were within the acceptable parameters, signifying the model to be fit.

**Table 4:** Model fit indices (SEM).

Fit index	Recommend values*	Observed values
Cmin/df	<3.0	4.194
GFI	0.90	0.886
AGFI	0.90	0.852
CFI	0.90	0.942
RMSEA	<0.070	0.081

*Source: Hair et al. (60), Hooper et al. (62), Hu and Bentler (63), and Malhotra and Dash (48).

Subjective norm positively impacts the intention to buy health insurance, leading to support hypothesis H2 (*ß* = 0.360; sig < 0.05). Similarly, hypothesis H3, depicting a positive relationship between perceived behavioral control and intention to buy health insurance, was also supported (*ß* = 0.364; sig < 0.05). Perceived product risk was also found to positively impact the intention to buy health insurance; thus, hypothesis H5 was supported (*ß* = 0.378; sig < 0.05). The relevant indices have been depicted in Table 5.

**Table 5:** Results of hypotheses testing.

	Estimate	C.R.	*p*
INHI 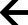 PERBC	0.364	6.770	***
INHI 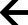 PPRsk	0.378	8.028	***
INHI 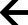 SNorm	0.360	5.680	***

***Significant (*p* < 0.05).

## 5 Discussion

India has been seeing constant economic growth and is one of the biggest economies in the world. Nearly 514 million individuals in India had health insurance coverage for the fiscal year 2021 (65). Government-sponsored health insurance programs covered most of them, while individual insurance policies covered the minority. The study findings suggest that subjective norms, perceived behavioral control, and perceived product risk (PERBC, PPRsk, and SNorms) positively impact the purchase intention for health insurance. In this study, we extend the TPB model by adding a new factor, i.e., perceived product risk. Thus, the study findings significantly contribute to the existing literature on health insurance. We took a cue from the extant literature and proposed a six-factor model. The proposed model comprises five independent factors that help determine the purchase intention of tobacco and alcohol consumers toward health insurance. However, the pilot study yielded three independent factors (SNorms, PERBC, and PPRsk) significantly influencing purchase intention. Attitude and perceived usefulness were removed from the scale because of low factor loadings and cross-loadings.

The findings of the present study indicate that tobacco and alcohol consumers in India are more concerned with subjective norms, perceived behavior control, and perceived product risk in the context of purchasing health insurance. The findings of this paper suggest that the three factors are essential and play a significant role in building up a positive purchase intention among tobacco and alcohol consumers toward health insurance. Moreover, to enhance the interest in obtaining health insurance among users of alcohol and tobacco, insurance policymakers and marketing practitioners should focus on these three factors.

The study findings align with the results of several researchers who found the above factors to have a significant role in determining consumers’ purchase intention. Bainchi et al. (32) suggested that consumer purchasing behavior is influenced by family, friends, relatives, colleagues, and others perceived as important. If the consumers can engage in any activity, they are more likely to buy it. The significance of perceived behavior control on purchase intention has been suggested by several researchers, including Photcharorn et al. (19) and Husin and Ab Rahman (35). The findings of our study also highlight the significance of perceived behavior control on the purchase intention of health insurance among tobacco and alcohol consumers. The study findings indicate that perceived product risk has a significant role in determining consumer purchase intentions, which aligns with the results of other researchers. Perceived product risk refers to the potential for a product to perform unconventionally compared to its original specification (45). When customers are uncertain about their choice, they experience risk during the purchasing process (46). According to the theory of how consumers perceive risk, customers feel risk when faced with uncertainty and unfavorable outcomes from actions that do not meet their expectations (46, 66, 67).

### 5.1 Implications

The study has examined the correlation between TPB components and the purchase intention of tobacco and alcohol consumers, leading to health insurance purchases. A new independent factor, perceived product risk (PPR), is embedded into the TPB model, which appears crucial for tobacco and alcohol consumers to purchase health insurance. In the present study, we extended the TPB model by incorporating perceived product risk to determine the purchase intention of health insurance among tobacco and alcohol consumers in India.

We have tried to explore the intention of a cohort rarely tapped by the research community. The study’s findings can help the insurance industry create strategies and make policies for the growth of their health insurance sector. Insurance companies should emphasize the unpredictability of the future to boost demand for health insurance. The policymakers of the insurance industry can incorporate subjective norms, perceived behavior control, and perceived product risk in their strategies to accelerate more demand for their health insurance products. In addition to encouraging individuals to get health insurance, the government and insurance providers may take the initiative to educate people on the value of having health insurance. This research makes a significant contribution to the field of health insurance studies. This study provides insurance companies and policymakers with a better understanding of consumer purchase behavior and their perception, which can be capitalized to enable them to be in the right direction. Based on the study findings, insurance companies should be able to develop more effective marketing strategies by focusing on tobacco and alcohol consumers.

Several behavioral factors, including insurance literacy, perceived complexity, and regulation literacy, influence the purchase intention among tobacco and alcohol consumers. This suggests that insurance providers must offer training courses to enlighten the public about health insurance, disseminate knowledge, and develop easier processes. Consumers would become more motivated to buy health insurance-related items and services.

### 5.2 Limitations and future directions

It should be kept in mind that our study has certain limitations. First, we have used an extended version of the TPB model in the current study. Future research could extend the model by including other relevant factors. Second, this study is related to the purchase intention toward health insurance among tobacco and alcohol consumers only. Third, the data represent respondents from a single host country (India), so the study findings may not apply to other parts of the world. Our study results should be validated by including respondents worldwide in future work.

## Data availability statement

The raw data supporting the conclusions of this article will be made available by the authors, without undue reservation.

## Ethics statement

Ethical approval was not required for the study involving humans in accordance with the local legislation and institutional requirements. The participants provided their written informed consent to participate in this study.

## Author contributions

AM: Conceptualization, Data curation, Formal analysis, Investigation, Methodology, Resources, Software, Visualization, Writing – original draft, Writing – review & editing. MJ: Conceptualization, Data curation, Formal analysis, Investigation, Methodology, Resources, Software, Validation, Writing – original draft, Writing – review & editing. AA: Conceptualization, Investigation, Methodology, Project administration, Resources, Software, Supervision, Validation, Writing – original draft, Writing – review & editing. SG: Investigation, Methodology, Resources, Validation, Writing – original draft, Writing – review & editing. DM: Project administration, Resources, Validation, Writing – review & editing.
